# Propagation topography of redox phase transformations in heterogeneous layered oxide cathode materials

**DOI:** 10.1038/s41467-018-05172-x

**Published:** 2018-07-18

**Authors:** Linqin Mu, Qingxi Yuan, Chixia Tian, Chenxi Wei, Kai Zhang, Jin Liu, Piero Pianetta, Marca M. Doeff, Yijin Liu, Feng Lin

**Affiliations:** 1 Department of Chemistry, Virginia Tech, Blacksburg, VA 24061 USA; 2 Beijing Synchrotron Radiation Facility, Institute of High Energy Physics, Chinese Academy of Sciences, Beijing, 100049 China; 3 Stanford Synchrotron Radiation Lightsource, SLAC National Accelerator Laboratory, Menlo Park, CA 94025 USA; 4 Department of Geological Sciences, Stanford University, Stanford, CA 94305 USA; 5 Energy Storage and Distributed Resources Division, Lawrence Berkeley National Laboratory, Berkeley, CA 94720 USA; 6 Department of Materials Science and Engineering, Virginia Tech, Blacksburg, VA 24061 USA

## Abstract

Redox phase transformations are relevant to a number of metrics pertaining to the electrochemical performance of batteries. These phase transformations deviate from and are more complicated than the conventional theory of phase nucleation and propagation, owing to simultaneous changes of cationic and anionic valence states as well as the polycrystalline nature of battery materials. Herein, we propose an integrative approach of mapping valence states and constructing chemical topographies to investigate the redox phase transformation in polycrystalline layered oxide cathode materials under thermal abuse conditions. We discover that, in addition to the three-dimensional heterogeneous phase transformation, there is a mesoscale evolution of local valence curvatures in valence state topographies. The relative probability of negative and positive local valence curvatures alternates during the layered-to-spinel/rocksalt phase transformation. The implementation of our method can potentially provide a universal approach to study phase transformation behaviors in battery materials and beyond.

## Abstract (web summary)

Here the authors demonstrate a spectroscopic and imaging approach to study redox solid-state phase transformation in lithium ion cathode materials under thermal abuse conditions. The valence curvature of the propagation front alternates as a result of local chemical and structural heterogeneities.

## Introduction

Solid-state phase transformations take place ubiquitously in crystalline materials when a parent phase becomes metastable due to an externally applied driving force, such as changes in temperature, pressure, electrical, or magnetic fields^1–6^. When a phase transformation is initiated, boundaries that separate the chemically and/or structurally distinct domains (i.e., phases) are spontaneously established^1,7–9^. These phase boundaries propagate as the phase transformation proceeds. How these solid-state phase transformations occur and propagate are fundamentally intriguing and determine behavior in many systems, including batteries^10–12^, structural materials^13^, and catalysts^14,15^. Solid-state phase transformation has been a subject of intense research in electrode and electrolyte inorganic battery materials, because the nucleation and development of secondary phases can govern the local electronic and ionic properties of these materials, thus impacting the battery behavior at the device level^16–19^. The knowledge obtained from directly observing phase transformation can improve our understanding of materials functionalities for practical battery applications^20–22^. Thus far, the direct visualization of solid-state phase transformation has been mostly limited to localized and discrete observation, namely, at the level of single-crystal particles or specific selected regions of a large particle ensemble^8,9,11,23,24^. However, phase transformations take place at multiple length scales and exhibit three-dimensional (3D) continuous patterns in polycrystalline heterogeneous ensembles (e.g., heterogeneous cathode secondary particles), which necessitates studies across different length scales ranging from atomic scale to mesoscale and macroscale. The rapid development of advanced analytical techniques, especially those associated with electron microscopy and synchrotron X-rays, has opened up vast opportunities for investigating phase transformation phenomena under non-equilibrium conditions over a wide range of time and length scales^25,26^. In redox active materials, phase transformation is commonly accompanied by redox reactions, and a phase boundary can be identified as the interface between the oxidized and reduced domains. Therefore, the visualization of phase transformation and propagation in these materials can be simplified to the spectroscopic determination of local electronic properties, such as valence states of elements participating in redox reactions. Some of our coauthors have recently studied phase transformations in transition metal oxide materials under non-equilibrium conditions with applied electrical fields and discovered that, in single-crystal metal oxides, phase propagation is highly dependent on the local defect density (e.g., line defects) and crystal orientation at the phase propagation fronts^10,11,24^. Meanwhile, a series of studies has been reported using synchrotron X-ray spectroscopic imaging to investigate phase transformations in LiFePO_4_^8,12,27,28^, LiCoO_2_^26,29^, and Li_x_Mn_1.5_Ni_0.5_O_4_^30^ materials. These studies implied that the nucleation and propagation of new phases determine the critical metrics of batteries, such as power density, energy density, and cycle life. Classical theories state that a solid-state phase transformation is initiated by forming spherically shaped nuclei with a critical domain size that is determined by the interfacial energy between the new phase and the parent phase as well as by the reduction of bulk free energy of the parent phase^31^. The subsequent phase propagation is rather anisotropic and determined by the crystallographic matching between the new phase and the parent phase. Ultimately, the new phase can be assembled into shapes that are distinct from those of nuclei. In a heterogeneous system, such as secondary polycrystalline ensembles of battery materials with many grain boundaries and defects, the phase propagation significantly deviates from the classical nucleation and propagation theories, as it can sporadically take place in chemically and structurally distinct locations at different length and time scales. Although most studies with model systems (e.g., big single crystals) can provide some insights into phase transformations in battery materials^12^, they fall short of what happens in real heterogeneous electrodes or polycrystalline particle ensembles. The mechanistic model is yet to be established to elucidate the solid-state phase transformation in these polycrystalline systems. A model of this kind is expected to bring insights into phase transformations in complex redox active materials, including battery electrodes and beyond.

Herein, we map the topography of phase propagation fronts in heterogeneous LiNi_x_Mn_y_Co_z_O_2_ polycrystalline ensembles by spectroscopically identifying the mesoscale 2D/3D distribution of the new phase and the parent phase. We then construct 3D topographies for the valence states of transition metal cations and discover that the phase propagation follows a complex pathway that is defined by the local valence curvature of valence state topographies. We anticipate that our model can potentially enable better understanding of solid-state phase transformations in redox active materials. Beyond batteries, our approach lays the ground for studying mesoscale behaviors of phase transformations in a broad range of redox active materials.

## Results

### 2D mapping of Ni valence states

The LiNi_0.4_Mn_0.4_Co_0.2_O_2_ (NMC) polycrystalline material (O3 layered structure with a space group of R$\bar 3$m) was chemically delithiated by nitronium tetrafluoroborate (NO_2_BF_4_) dissolved in acetonitrile (CH_3_CN) to obtain chemically delithiated Li_0.4_Ni_0.4_Mn_0.4_Co_0.2_O_2_^32^. During this process, nickel cations are oxidized above the Ni^2+^ valence state and oxygen anions are activated through the Ni*3d*–O*2p* hybridization^9,32–34^. Upon heating, the delithiated Li_0.4_Ni_0.4_Mn_0.4_Co_0.2_O_2_ material undergoes a phase transformation from the R$\bar 3$m layered structure to a mixed spinel/rocksalt structure with simultaneous reduction of nickel, manganese, and cobalt cations and release of molecular oxygen^16,32,35^. We note that such a phase transformation has been well demonstrated in layered cathode materials under thermal abuse conditions^16,17^, which provides a good knowledge basis for the present study. Previously, we investigated phase transformation at the nanoscale using transmission electron microscopy and observed the heterogeneous nature of the transformation defined by crystal defects such as grain boundaries^10^. The origins of the heterogeneous phase transformation are attributable to the heterogeneous distribution of transition metals, the anisotropic volume change, oxygen release, formation of cracks, grain boundaries, and the random orientation of primary particles^10,20,36^. To expand such a localized study to the mesoscale in polycrystalline materials, in this study we applied in operando full-field transmission X-ray microscopy (TXM), which can concurrently monitor a large number of phase transformation events allowing us to statistically construct 2D contour maps and 3D topographies for phase propagation fronts in large particle ensembles. The 2D imaging contains depth-averaged information, potentially causing difficulty for studying of fine features. In contrast, 3D data make it possible to extract the chemical information at a certain desired depth. However, the amount of time required by the 3D XANES (X-ray absorption near edge spectroscopy) data acquisition makes it challenging to perform in operando 3D XANES mapping within a reasonable time frame. As a result, the rational design of our experiment becomes to perform 2D XANES mapping in operando and then conduct a 3D XANES mapping at the end of our experiment to capture the final state in more details. We also designed our experiment in a way that the “final state” was not fully reacted. As a result, we can visualize the 3D chemical heterogeneity, which serves as the chemical basis for developing the chemical curvature. Furthermore, we have performed thorough experiments to justify our method of studying phase transformation using in operando 2D TXM (Supplementary Information).

**Fig. 1:**
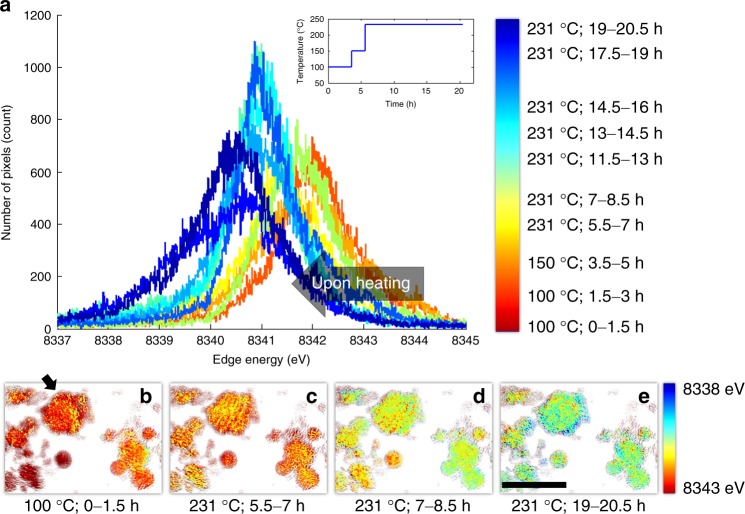
Direct visualization of the changing Ni valence state in Li_0.4_Ni_0.4_Mn_0.4_Co_0.2_O_2_ polycrystalline ensembles upon in situ heating. **a** The histogram of the local valence states of Ni (represented by the Ni K-edge X-ray absorption spectroscopy (XAS) absorption edge) over the entire field of view, with the heating pattern shown in the inset. **b–e** The maps of the Ni valence states observed in situ, which are color coded to the local Ni valence state as indicated by the color map to the right of (**e**). The Ni valence states are represented by the Ni K-edge XAS absorption edge, where a higher energy and a lower edge energy represent a higher and a lower Ni valence states, respectively

As the polycrystalline ensembles were heated, there was a continuous overall reduction of nickel cations, which was time and temperature dependent (i.e., peaks shifted to lower energy in Fig. 1a). The spatially resolved Ni K-edge XANES method had an energy resolution of ~1 eV (Supplementary Figure 1), which is smaller than the peak width of XANES spectral histogram in Fig. 1a. Therefore, the broad energy distribution of the Ni K-edge energy in Fig. 1a implies that the distribution of Ni valence states was highly heterogeneous, which was also confirmed by the 2D projection maps of Ni valence states (Fig. 1b–e). We also note that the energy range in Fig. 1a is slightly larger than that one would expect, possibly due to the limited signal to noise ratio in the spectra associated with every single pixel at ~30 nm. In these large polycrystalline ensembles, the nucleation of the spinel/rocksalt phase was sporadic and proceeded throughout the entire period of heating from 100 to 231 °C. The broad distribution of nickel valence state (Fig. 1a) implies that the bulk layered-to-spinel/rocksalt transformation is not complete even at 231 °C, which is consistent with a previous study^16^. The heterogeneity of Ni valence states can be attributed to different degrees of oxygen release, which could therefore serve as a key indicator for quantifying the degree of phase transformation that occurs at the corresponding location. We are aware that a thorough quantification of oxygen release could potentially better justify the processing of phase transformation using nickel valence state. Nevertheless, since O2p is primarily hybridized with Ni3d in delithiated NMCs^34^, the oxygen release is mostly influenced by Ni valence states, thus justifying our method. Furthermore, our simplified processing method is supported by our recent in situ transmission electron microscopy studies^32^ and those reported in the literature^25,37,38^. We conjecture that the spinel/rocksalt phases might be populated at the surfaces of primary particles or along grain boundaries.

**Fig. 2:**
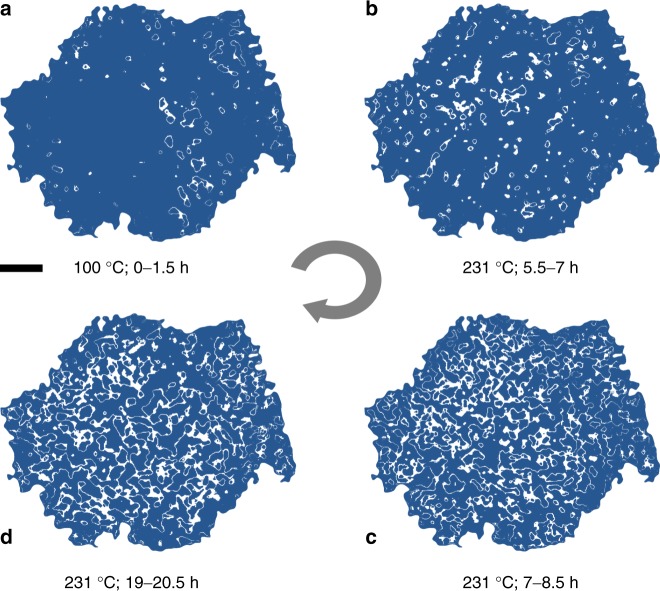
The development of the Ni reduction reaction front upon heating. The white pixels in the map are the pixels that have Ni K-edge position at 8341 ± 0.1 eV. The blue area in the figure is associated with local Ni K-edge position outside of the defined Ni K-edge energy window. **a** is the reaction front map over the particle after it is heated for up to 1.5 hours at 100 °C. **b**-**d** are the reaction front maps over the particle after it is heated at 231 °C for up to 7, 8.5, and 20.5 hours, respectively.The scale bar is 2 μm

To gain deeper insight into the spatial evolution of the phase transformation at mesoscale, we studied 2D Ni valence state maps over the particle indicated by the black arrow in Fig. 1b in more detail. We segmented the Ni valence state maps and labeled the pixels with Ni K-edge positions at 8341 ± 0.1 eV (white pixels in Fig. 2) as the active reaction front. The edge energy value 8341 eV was chosen because it is at the middle of the overall distribution of the local Ni valence states in Fig. 1, which could be interpreted as halfway transformed. We observed that nucleation events occurred sporadically at the early stages of the reaction as shown in Fig. 2a, b, where the active reaction regions are highlighted in white. The reaction front further developed into a complex interconnected network in Fig. 2c, d. These 2D contour maps provide the first evidence of complex pathways for the redox phase transformation in heterogeneous polycrystalline ensembles. We noticed that a good amount of fine features at ~100–200 nm is observed in our operando 2D chemical maps (Figs. 1b–e and 2). Although the 2D measurement loses the depth information by averaging along the beam path, it still provides sufficient 2D spatial resolution at ~30 nm, revealing the lateral chemical heterogeneity, which is an important piece of information and has offered critical insights into the understanding of the degradation, relaxation^39^, and phase transformation mechanisms^26,40–42^; in battery materials. Without the depth information, it is, however, challenging to perform more impactful quantification of the behavior of reaction fronts using 2D Ni valence maps. The lack of depth information hinders the attempt to link the 2D chemical features to the particle’s morphological structure, which is hierarchically complex in 3D. As a result, we do not attempt to conduct more comprehensive quantification of the reaction fronts in 2D. Our interpretation of the operando 2D valence maps is kept at a level that is robust to the debate on the validation of 2D/3D measurements (Supplementary Figures 2, 3). The reaction initiates at separated domains and propagates throughout the particle, which does not rely on a few high resolution features and is robust against the statistical error.

### 3D mapping of Ni valence states and topography

**Fig. 3:**
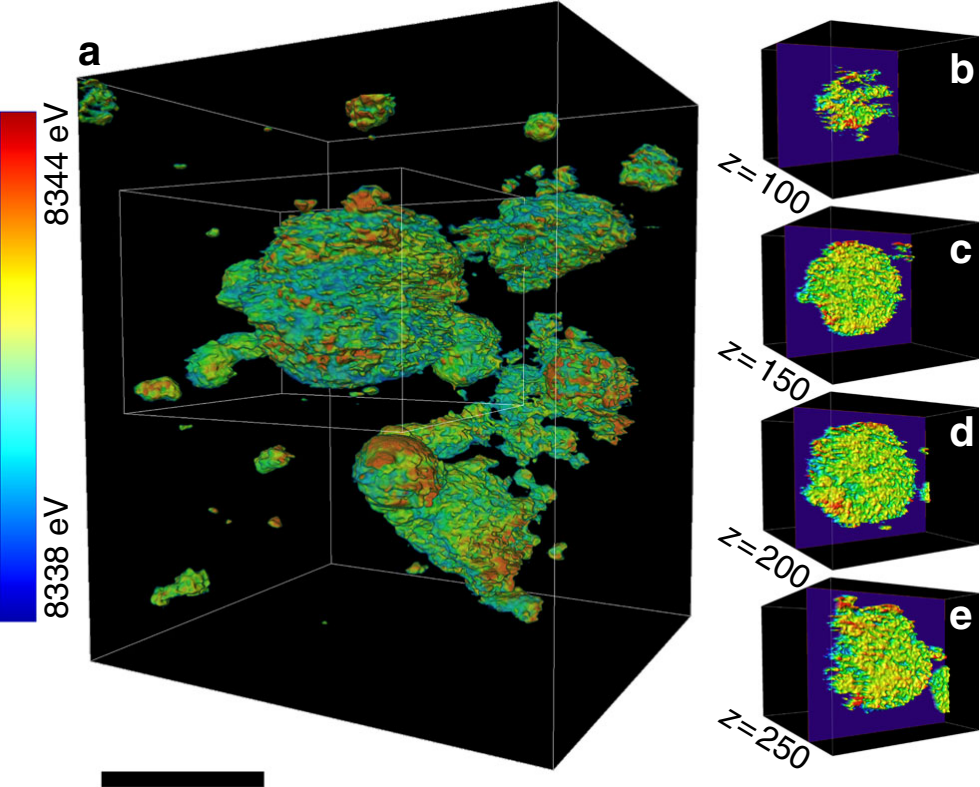
Three-dimensional distribution of Ni valence states over the polycrystalline ensembles after the heating sequence illustrated in Fig. 1a. **a** The 3D distribution of nickel valence states represented by Ni K-edge energy. The single-voxel thick slices through different depths of a particle (30 nm thick slices at 50 voxels apart through the largest particle in **a** are shown in **b-e**. The scale bar is 8 μm

To overcome the limitations in the 2D investigation, we reconstructed 3D maps for nickel valence states over the same polycrystalline ensembles after heating at 231 °C (Fig. 3). The 2D slices through different depths of the selected particle are shown in Fig. 3b–e, which highlight the 3D heterogeneity of Ni valence states throughout the bulk. This observation is consistent with the 2D heterogeneity reported in Fig. 1.

**Fig. 4:**
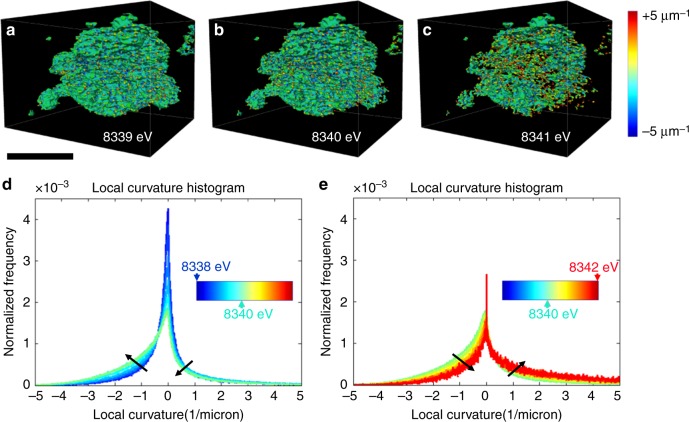
Evolution of local valence curvatures upon the phase transformation. Isosurfaces of the 3D Ni valence state maps at **a** 8339 eV, **b** 8340 eV, and **c** 8341 eV. The surface is color coded to the local valence curvature, with red and blue denoting the extremes of positive and negative curvature, respectively. **d**, **e** The histograms for the changes of local valence curvatures as a function of the selected energy value **d** from 8338 to 8340 eV and **e** from 8340 to 8342 eV are shown. The scale bar is 10 μm

It is evident that the heterogeneous nature of phase transformation prevails, independent of the ensemble size, in all of the polycrystalline ensembles in 3D. The phase transformation followed complex pathways that are likely influenced by grain boundaries and local chemical environments such as compositional variation and defects. The regions surrounding defects are usually more reactive and may redirect the propagation pathway of the spinel/rocksalt phases^32^. As discussed earlier, the local Ni valence state serves as an indicator for quantifying the degree of the phase transformation. The 3D topographies of Ni cations at various valence states from the relatively more reduced (8339 eV Ni K-edge) to the more oxidized (8341 eV Ni K-edge) provide a direct visualization of the 3D propagation fronts, which is shown in Fig. 4. The reduced (lower Ni K-edge energy) and oxidized (higher Ni K-edge energy) represent the new and parent phases, respectively. The propagation of the reaction front proceeds from Fig. 4a (more reduced isosurface where the phase transformation already occurred, new phase) to Fig. 4c (more oxidized isosurface where the phase transformation is yet to occur, parent phase). In the reductive phase transformation (this study), the phase transformation propagates from the new (reduced) phase into the parent (oxidized) phase. We calculated the local valence curvatures over the isosurface of each topography and color coded the isosurfaces (Fig. 4a–c) based on the corresponding local valence curvature, with the red and blue indicating positive and negative local curvatures, respectively. Although we only display the isosurfaces at three discrete energy points, the graduated propagation of the reaction front can be investigated by tuning the energy value in finer steps. This allows us to study the histogram of local valence curvatures, which shows the curvature probability distribution changes as a function of the selected energy value (Fig. 4d, e).

Interestingly, we discovered that the histogram of local valence curvatures at the reaction front underwent oscillation as a function of Ni valence states, as indicated by the black arrows in Fig. 4d, e. When the phase propagation proceeded from the 8338 eV Ni K-edge isosurface to the 8340 eV Ni K-edge isosurface, a major increased probability of negative curvatures was observed, and the probability of positive curvatures slightly decreased (Fig. 4d). However, when phase propagation proceeded from the 8340 eV Ni K-edge isosurface to the 8342 eV Ni K-edge isosurface, the probability of negative and positive curvatures experienced a major decrease and a slight increase, respectively (Fig. 4e).

**Fig. 5:**
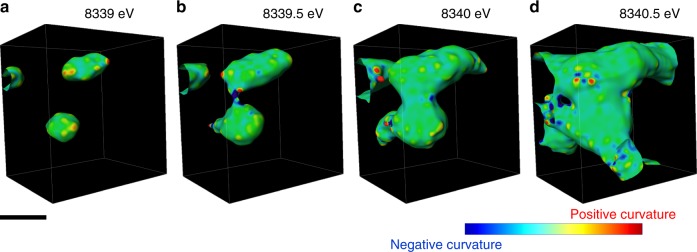
Visualization of the phase front propagation at a selected localized region. The isosurface develops as thresholding Ni K-edge energy value is changed: **a** 8339 eV, **b** 8339.5 eV, **c** 8340 eV, and **d** 8340.5 eV. The evolution of the isosurface shows the propagation of the reaction front of the Ni reduction upon heating. The isosurface is color coded to the local curvature with red and blue showing positive and negative curvatures, respectively. The white arrow in (**b**) points to a neck region (see text). The scale bar is 1 μm

When we divide the propagation into segments, we can clearly visualize the oscillation (Fig. 5). For example, we selected a localized region to visualize the evolution of valence curvatures as a function of Ni K-edge energy. At the low Ni K-edge energy (i.e., 8339 eV), there were discrete sites with mostly positive curvatures, indicating the events of sporadic heterogeneous nucleation. Subsequently, as the phase propagation proceeded to the intermediate Ni K-edge energy (i.e., 8339.5 eV), the discrete phase transformation sites aggregated and formed neck-like structures (arrow in the Fig. 5b), where extremely negative local curvatures were populated, suggesting the merging of new domains. As the phase propagation proceeded to the higher energy region (8340 and 8340.5 eV), the neck region grew and ultimately disappeared. Subsequently, the concentration of negative curvatures at the neck region decreased, leaving behind mostly neutral or positive curvatures (Fig. 5c, d). We conjecture that such a heterogeneous phase transformation behavior was originated from the heterogeneous phase transformation at the nanometric scale that was recently reported for this material using in situ transmission electron microscopy. The initiation of the phase transformation took place more rapidly at the surface and along the grain boundaries^32^.

## Discussion

The chemical and structural stability of cathode materials under heating conditions have strong implications for battery safety. Chemical and structural transformations usually accompany redox reactions and oxygen release, which can induce severe thermal runaway, and even catastrophic failure of rechargeable batteries with flammable organic electrolytic solutions. Previous studies have shown that oxygen release and phase transformation of battery materials occur under thermal abuse conditions^16,17^, but fundamental studies in large battery ensembles have been limited to model systems, such as single crystals. Our study goes beyond model systems and creates a new methodology to study phase transformations in polycrystalline and heterogeneous particle ensembles under thermal abuse conditions. Moreover, our study may have profound implications for addressing other important scientific questions in batteries, as discussed below.

Redox reaction-induced phase transformations are an important subject in solid-state phase transformation and determine the reaction kinetics in electroactive materials. Our study proposes a conceptually new method to investigate phase nucleation and propagation in polycrystalline battery electrode materials. The physical measurement and mathematical processing of transition metal X-ray absorption edges allow for tracking the 3D pathway of phase propagation. We believe that future studies can take advantage of this method to address a number of fundamental questions in battery science. Here we discuss a few potential applications of this method. First of all, we anticipate that the method developed herein could be applied to study battery cycling under operating conditions to obtain a more sophisticated understanding of phase propagation, which takes into account the considerable heterogeneities encountered in electrodes. The method is based on mapping valence states, hence any redox reaction in solid-state electrode materials can in theory be studied using the same methodology. There have been several studies demonstrating the charge heterogeneity in electrode materials^9,39,43^, and the charge heterogeneity greatly influences the rate capability of batteries. It is possible to study the origin of the charge heterogeneity in 3D and in real time. Secondly, the phase propagation pathway is likely impacted by the local chemical and structural environments. If we can obtain knowledge on how the interaction between the phase propagation and the local environment evolves, we can make use of the 3D phase propagation topography to inform the design of chemical and structural properties that can accelerate the kinetics of favorable phase transformation during battery usage. Last but not least, many failure modes of battery materials involve undesired redox reaction-induced phase transformations, which create electrochemically inert phases, for example, the formation of rocksalt materials on layered oxide cathode materials^10,44^, and the oxygen evolution-induced phase transformation in lithium/manganese-rich layered oxide materials^35^. Understanding the 3D distribution of these inert phases and how they propagate in large battery polycrystalline ensembles can provide insights into developing methods to suppress undesired phase transformations. The experimental results suggest the importance of designing efficient architecture of secondary particles. With the efficient construction of NMC secondary particles, good controls of anisotropic strain, cracks, and heterogeneous phase transformations under abuse conditions would be better realized. Ultimately, the inhibition of phase transformations can potentially implant improved thermal stability.

In summary, we have demonstrated a conceptually new approach to study redox solid-state phase transformation in heterogeneous and polycrystalline layered oxide cathode materials under thermal abuse conditions. Our model polycrystalline system (i.e., charged layered cathode material) has revealed important characteristics that were previously impossible to probe. At the mesoscale, the phase transformation from the oxidized (layered) to the reduced phases (mixed spinel/rocksalt) follows highly complex 3D pathways. There are changes in the local valence curvatures of the topographies constructed by using transition metal cations with identical valence states, oscillating between positive and negative curvatures. Such a change of local valence curvatures is associated with the heterogeneous and discrete nucleation of new phases as well as possibly with the local chemical and structural heterogeneity (e.g., compositional variation and grain boundaries), which warrants our future studies. The present study improves our understanding of phase transformation in battery materials and lays the ground for studying charging and discharging kinetics of batteries using 2D contour maps and 3D topographies. It is important to note that practical batteries under thermal abuse conditions, with the presence of liquid electrolyte, can potentially be studied using the methodology developed in this study. However, if the single-particle resolution is needed, the TXM technique needs to be improved to collect data at a much faster rate, because thermal abuse in practical batteries usually takes place much more rapidly and the resulting temperature is uncontrollable. In addition, the presence of liquid electrolyte could potentially shift the particles undergoing measurement, thus one would need to improve the particle tracking and involve sophisticated image alignment in the data processing. Finally, we anticipate that our methodology is applicable to redox phase transformations far beyond battery systems that are polycrystalline in nature.

## Methods

### Preparation of materials

LiNi_0.4_Mn_0.4_Co_0.2_O_2_ was synthesized by a co-precipitation method with NiSO_4_•6H_2_O (Sigma Aldrich, 99.99%), MnSO_4_•H_2_O (Sigma Aldrich, 99%), and CoSO_4_•7H_2_O (Sigma Aldrich, 99%) as the starting precursors. The transition metal solution (0.04 M NiSO_4_•6H_2_O, 0.04 M MnSO_4_•H_2_O, and 0.02 M CoSO_4_•7H_2_O dissolved in 100 mL H_2_O), starting solution (40 mL NaOH and NH_3_•H_2_O aqueous solution with a molar ratio NaOH/NH_3_ = 1.2, pH value is adjusted to 10.5), and base solution (100 mL NaOH and NH_3_•H_2_O aqueous solution with a molar ratio NaOH/NH_3_ = 1.2) were made and separately stored in Kimble bottles under N_2_ protection. The transition metal solution and base solution were simultaneously pumped into the starting solution at a feed rate of ~ 2 mL min^−1^ with continuously stirring at 50 °C. Initially, the feed rate of the base solution was frequently tuned to allow the reaction to take place at pH 10.5 ± 0.2, until the pH stabilized. The precipitate was collected, washed, and filtrated with deionized water and dried in vacuum oven overnight at 100 °C. The dried precursor was then mixed with LiOH thoroughly and calcined under air flow (2 L min^−1^) at 725 °C for 6 h to obtain the final LiNi_0.4_Mn_0.4_Co_0.2_O_2_ powder. Subsequently, delithiated Li_0.4_Ni_0.4_Mn_0.4_Co_0.2_O_2_ particles were obtained by chemical delithiating LiNi_0.4_Mn_0.4_Co_0.2_O_2_. Briefly, LiNi_0.4_Mn_0.4_Co_0.2_O_2_ powder was dispersed in 0.11 M NO_2_BF_4_ (Acros Organic, 97%) dissolved in acetonitrile (CH_3_CN, Fisher Chemical, 99.9%), and continuously stirred for 24 h in an Ar-filled glovebox (H_2_O < 0.5 ppm, O_2_ < 0.5 ppm) at room temperature. Then, the delithiated powder was collected and washed for three times using acetonitrile. Finally, the Li_0.4_Ni_0.4_Mn_0.4_Co_0.2_O_2_ product was obtained by overnight vacuum drying.

### In situ imaging and data processing

We conducted in situ X-ray spectro-microscopic scan of the Li_0.4_Ni_0.4_Mn_0.4_Co_0.2_O_2_ particles using the TXM at beamline 6-2C of Stanford Synchrotron Radiation Lightsource of the SLAC National Accelerator Laboratory. The powder sample was loaded into a quartz capillary (100 µm in diameter and 10 µm in wall thickness) for in situ imaging under precise temperature control using an in-house developed heater that is compatible with the TXM setup. The typical exposure time for single images is 0.5 s. The spatial resolution of this instrument is ~30 nm. More details of the synchrotron beamline configuration and the concept of X-ray spectro-microscopy and spectro-tomography can be found elsewhere^45,46^. In the 2D spectro-microscopic scan, the energy of the incident X-rays is scanned from 6390 to 8600 eV covering the absorption K-edges of Mn, Co, and Ni. Only the data over the Ni K-edge are presented and discussed in this work because it underwent the most significant changes of valence state. In the 3D XANES scan, tomography was performed at 53 different energy points over the desired energy window (8200–8450 eV). In the near edge region (8325–8360 eV), we chose the energy step at 1 eV to ensure sufficient energy resolution. The pre-edge and post-edge regions were scanned with larger energy steps of 20 eV to cover a relatively wide energy window for normalization of the spectra. The TXM data processing was performed using an in-house developed software package known as TXM-Wizard^45^. To calculate the chemical curvature, we first built a topographic isosurface for Ni K-edge with an identical energy, and then we calculate the curvature with a spatial resolution of 30 nm on the isosurface.

### Data availability

The authors declare that all the relevant data are available within the paper and its Supplementary Information file or from the corresponding author upon reasonable request.

## Electronic supplementary material

Supplementary Information
