# Supplementary material for: Propagation topography of redox phase transformations in heterogeneous layered oxide cathode materials
Source: Nat Commun. 2018 Jul 18;9:2810. doi: 10.1038/s41467-018-05172-x (PMC6052144; doi:10.1038/s41467-018-05172-x)
Supplement: Supplementary file 1 — Supplementary Information [file 41467_2018_5172_MOESM1_ESM.pdf]

Propagation Topography of Redox Phase Transformations in Heterogeneous Layered Oxide Cathode Materials

Mu et al

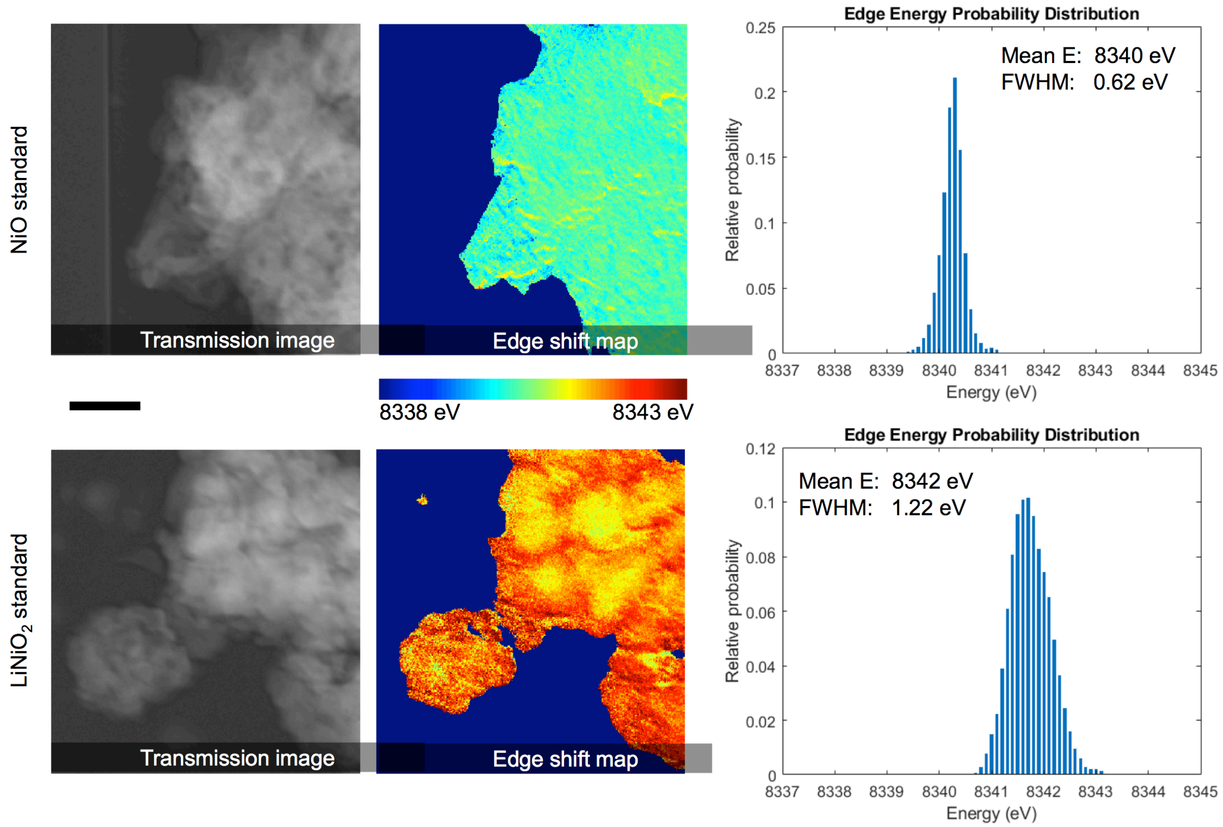

Supplementary Figure 1. Transmission images, edge shift maps and the corresponding edge energy probability distribution plots of standard Ni compounds, NiO and $LiNiO_2$. The scale bar is 3 μm.

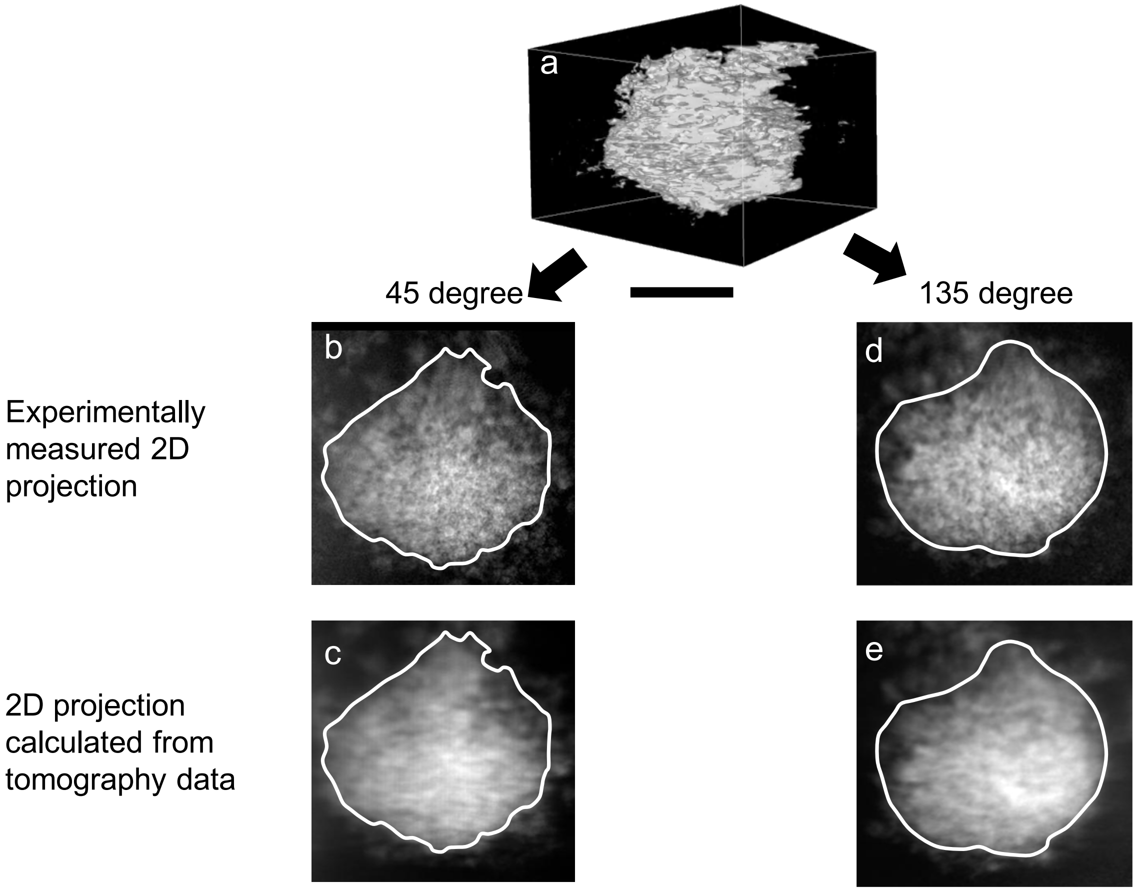

Supplementary Figure 2. Single energy tomographic result is shown in panel a. Panels b and d are experimentally measured projection images in two different viewing angles. Panels c and e are the projection images calculated from the 3D data in the corresponding viewing angles. The scale bar is 6 μm.

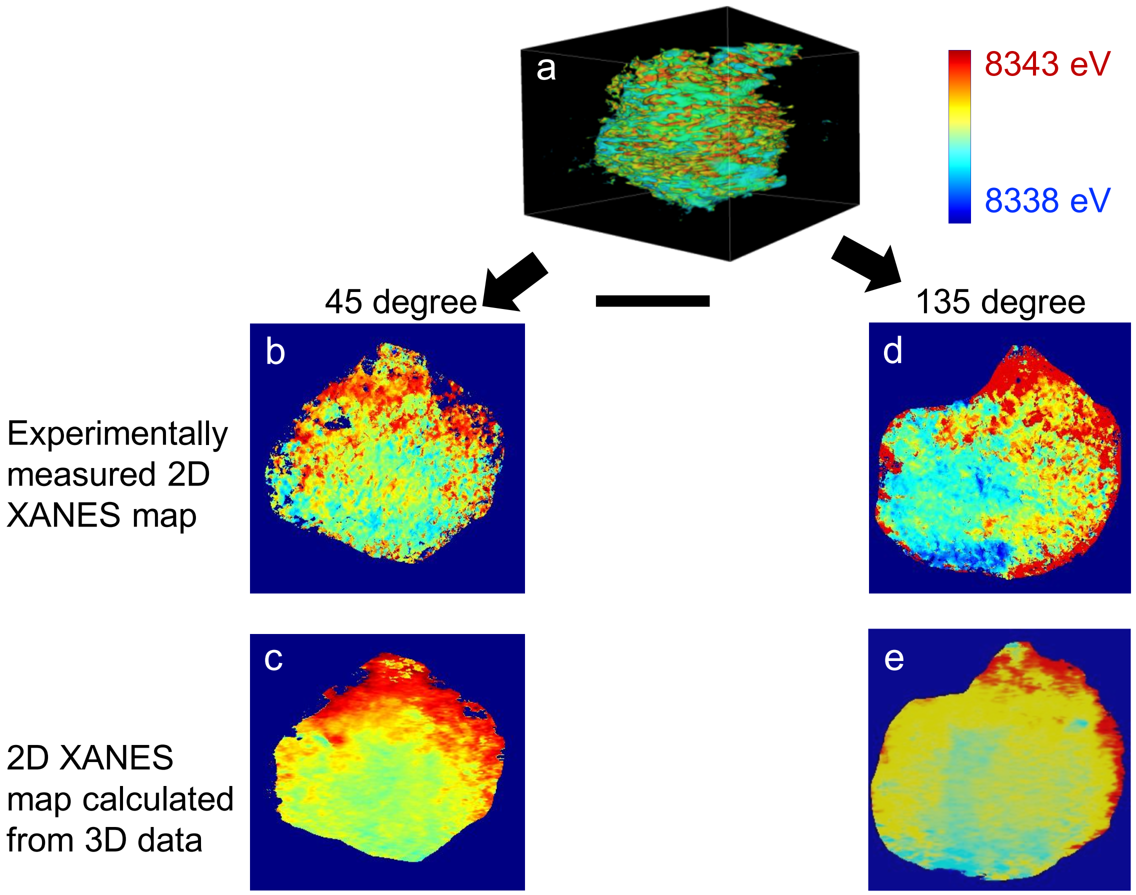

Supplementary Figure 3. 3D XANES mapping result is shown in panel a. Panels b and d are experimentally measured 2D XANES map in two different viewing angles. Panels c and e are the projection 2D XANES maps calculated from the 3D data in the corresponding viewing angles. The scale bar is 6 μm.

Supplementary Note 1

In order to demonstrate the instrumental energy resolution, we have performed additional measurements of standard materials using the same experimental configuration (Supplementary Figure 1). Standards of $Ni^{2+}O$ and $LiNi^{3+}O_2$ show clearly shift in the edge energy distribution due to the difference in the corresponding valence state of Ni. The color coded edge energy maps and the corresponding histograms in the right column of Supplementary Figure 1 suggest that the mean values of the edge energy of nickel species in NiO and $LiNiO_2$ are 8340 eV and 8342, respectively. The FWHM of the NiO standard is only 0.62 eV, which is at the energy resolution defined by the monochromator of this beamline at SSRL, while the value of $LiNiO_2$ is about a factor of 2 larger. The broadening of the histogram of $LiNiO_2$ is possibly caused by the Li-Ni cation mixing of the standard sample. It should be noted, however, the Li-Ni cation mixing is evitable for this material.[1] With this experimental evidence, we are confident to claim an energy resolution of ~1 eV. The histogram shown in Figure 1a is clearly broader than our energy resolution.

In ideal scenario, the projection data calculated from the 3D data should be identical to the 2D measurement in the same viewing angle. Hold this in mind, we conducted more experiment and analysis for a systematic evaluation. Before we investigate the chemical states, an evaluation of single energy imaging data could be insightful. As shown in Supplementary Figure 2, we conducted the tomographic reconstruction of a selected particle. Both the experimentally measured projection images (b and d in Supplementary Figure 2) and the ones calculated from 3D data (c and e in Supplementary Figure 2) are shown in the corresponding panels. We also chose to perform the evaluation in two different viewing angles at 90 degrees apart to ensure that our arguments are generally applicable. As shown in the experimentally recorded projection images (b–e in Supplementary Figure 2), features at primary particle level are clearly visible even though it is averaged through the depth of particle (~10 microns). The 2D projection images calculated from the 3D data (Supplementary Figures 2c and 2e) are in agreement with the experimentally measured projection images (Supplementary Figures 2b and 2d). However, it is clearly observed that the fine features are not fully preserved in Supplementary Figures 2c and 2e. This is because the alignment of projections images for tomographic reconstruction is not trivial at this resolution. When doing nanoscale x-ray tomography, the motor error become clearly visible, leading to a noticeable point spread function (PSF) even after manual/auto correction of the projection images. The PSF in the

3D reconstruction is further amplified when doing numerical simulation of the projection, resulting in blurred projection images as shown in Supplementary Figures 2c and 2e.

We then performed the same analysis on the 3D XANES. We present in Supplementary Figure 3, the 3D XANES result as well as the 2D XANES maps in the same manner as that of Supplementary Figure 2. We observed the general agreement between Supplementary Figures 3b and 3c, as well as 3d and 3e, confirming that the valance state mapping is reliable. The fine features are again not fully preserved in both Figures 2c and 2e, in part due to the same reason we discussed above for the single energy tomography data.

We would point out that some of the very fine features in Supplementary Figures 3b and 3d could also be resulted from the insufficient signal to noise in our data, because the determination of the edge energy is not very robust against the noise. In this context, the 3D XANES data has another advantage because it is built up through the reconstruction of projection images in many different viewing angles. The tomographic reconstruction procedure can be considered as an accumulation of the signals, although the motor error could lead to reduction in the spatial resolution in our case. Nevertheless, the overall agreement between the Supplementary Figures 3b to 3e, confirms the reliability of our experiment and analysis. There are pros and cons of both the 2D and 3D approach, but the overall trend suggested by the final results remains the same.

Supplementary Reference

1. Xu J, Lin F, Doeff MM, Tong W. A review of Ni-based layered oxides for rechargeable Li-ion batteries. *J Mater Chem A* **5**, 874-901 (2017).
